# Rhythmic Regulation of DNA Methylation Factors and Core-Clock Genes in Brain Structures Activated by Cocaine or Sucrose: Potential Role of Chromatin Remodeling

**DOI:** 10.3390/genes12081195

**Published:** 2021-07-31

**Authors:** Lamis Saad, Andries Kalsbeek, Jean Zwiller, Patrick Anglard

**Affiliations:** 1 Laboratoire de Neurosciences Cognitives et Adaptatives (LNCA), UMR 7364 CNRS, Université de Strasbourg, Neuropôle de Strasbourg, 67000 Strasbourg, France; lamisaad_8@hotmail.com (L.S.); zwiller@neuro-cnrs.unistra.fr (J.Z.); 2 The Netherlands Institute for Neuroscience (NIN), Royal Netherlands Academy of Arts and Sciences (KNAW), 1105 BA Amsterdam, The Netherlands; 3 Department of Endocrinology and Metabolism, Amsterdam UMC, University of Amsterdam, 1066 EA Amsterdam, The Netherlands; 4 CNRS, Centre National de la Recherche Scientifique, 75016 Paris, France; 5 INSERM, Institut National de la Santé et de la Recherche Médicale, 75013 Paris, France

**Keywords:** cocaine, sucrose, DNA methylation, circadian rhythms, appetite and satiety, addiction, neuroepigenetics

## Abstract

The circadian system interacts with the mesocorticolimbic reward system to modulate reward and memory in a time-of-day dependent manner. The circadian discrimination of reward, however, remains difficult to address between natural reinforcers and drugs of abuse. Circadian rhythms control cocaine sensitization and conversely cocaine causes long-term alteration in circadian periodicity in part through the serotonergic neurotransmission. Since neural circuits activated by cocaine and natural reinforcers do not completely overlap, we compared the effect of cocaine with that of sucrose, a strong reinforcer in rodents, by using passive chronic administration. The expression of fifteen genes playing a major role in DNA methylation (*Dnmts*, *Tets*), circadian rhythms (*Clock*, *Bmal1*, *Per1/2*, *Cry1/2*, *Rev-Erbβ*, *Dbp1)*, appetite, and satiety (*Orexin*, *Npy*) was analyzed in dopamine projection areas like the prefrontal cortex, the caudate putamen, and the hypothalamus interconnected with the reward system. The corresponding proteins of two genes (Orexin, Per2) were examined by IHC. For many factors controlling biological and cognitive functions, striking opposite responses were found between the two reinforcers, notably for genes controlling DNA methylation/demethylation processes and in global DNA methylation involved in chromatin remodeling. The data are consistent with a repression of critical core-clock genes by cocaine, suggesting that, consequently, both agents differentially modulate day/night cycles. Whether observed cocaine and sucrose-induced changes in DNA methylation in a time dependent manner are long lasting or contribute to the establishment of addiction requires further neuroepigenetic investigation. Understanding the mechanisms dissociating drugs of abuse from natural reinforcers remains a prerequisite for the design of selective therapeutic tools for compulsive behaviors.

## 1. Introduction

Drugs of abuse and natural reinforcers such as food or sugar activate some common brain structures of the mesocorticolimbic reward system and can lead to addiction characterized by a compulsive behavior despite the awareness of negative effects. However, functional magnetic resonance imaging [1], as well as electrophysiological [2,3] and molecular studies [4,5] have shown that their activated neural circuits do not completely overlap. How they differ in triggering various behaviors remains an open issue. Drugs of abuse have been shown to exert their action, at least partially, by regulating epigenetic mechanisms controlling transcription. In an earlier study, we showed that cocaine was modulating the expression of epigenetic factors, highlighting serotonin-elevating agents as a major component in neurotransmission involved in this modulation and in chromatin remodeling [6,7], as in other drugs of abuse [8]. Among these factors, the regulation of methyl-CpG-binding proteins MeCP2 considered as a major epigenetic factor [9] and MBD1 suggested that post-mitotic neurons could reinterpret the DNA methylation code they acquired during early development. Moreover, cocaine was found to regulate DNA methyltransferases (Dnmts) and ten-eleven translocation (Tets) methylcytosine dioxygenases expression controlling DNA methylation and demethylation processes [5,10,11]. Consistent with these findings, DNA methylation of genes such as the cyclin-dependent kinase-like 5 [12] involved in the “early-onset seizure” variant of Rett syndrome [13], the memory suppressor gene protein phosphatase-1 [11,14], Homer2 regulating glutamate signaling and synaptic plasticity [15] and the Orexin receptor-1 gene [5] regulating the reinforcing and rewarding properties of cocaine [16] has been reported to be modulated by cocaine. In addition, genome-wide DNA methylation studies support a crucial role of DNA methylation in drug-induced behaviors [17,18,19,20]. It is now clear that DNA methylation is not restricted to behaviors including addiction, but also plays a prominent role in learning and memory processes [21], in neurodevelopmental-associated disorders [22], or in diet choice [23], and developmental timing [24]. The circadian system interacts with the mesocorticolimbic reward system to modulate reward and memory in a time-of-day dependent manner [25] although the circadian discrimination of reward still remains difficult to address between drugs of abuse and natural reinforcers, as previously reported [26]. Cocaine sensitization and reward are under the control of circadian genes and rhythms [27] and conversely, cocaine causes long-term alteration in circadian periodicity [28] in part through the serotonergic neurotransmission [29,30]. Dynamic DNA methylation changes in circadian clock genes also result from clock misregulation in cancer [31] and changes in circadian period length have been shown to require de novo DNA methylation [32]. On the other hand, methyl donors and Dnmt inhibitors regulate cocaine sensitization and self-administration [33,34,35,36] similar to orexin-receptor antagonists inhibiting cocaine-induced behavior in preclinical trials [37,38,39,40]. A few studies have also provided evidence for the regulation of orexin and of their receptors by DNA methylation in embryonic stem cells, in cancer, in depressive disorders and in response to drugs of abuse [5,41,42,43,44]. As recently reported, the orexin system function extends beyond general reward seeking and plays a critical role in expression of the multiphenotype addiction-like state [45].

In the present study, we compare the effects of repeated cocaine intake with that of sucrose on genes and proteins playing a major role in DNA methylation, circadian rhythms, appetite, and satiety. Our data highlight major differences in the mechanisms by which cocaine and sucrose, a strong natural reinforcer in rodents, differ in triggering molecular and cellular changes in rat dopaminergic brain projection areas and in the hypothalamus.

## 2. Materials and Methods

### 2.1. Animals

Male Wistar rats, 7–8 weeks old at their arrival in the laboratory and weighting 160–180 g, were housed in standard home cages in a temperature and humidity-controlled room with a 12 h/12 h light/dark cycle with lights on at 7.00 am (Zeitgeber time ZT0) and lights off at 7 p.m. (ZT12). ZT is a standardized 24-h notation of the phase in which ZT0 indicates the beginning of light phase and ZT12 the beginning of the dark phase. All rats were allowed to acclimate to laboratory conditions and were handled during one week before experimental procedures started and had ad libitum access to food and water. They were then treated by cocaine hydrochloride i.p. injections (20 mg/kg) or by sucrose (1 mL/15% solution) passive oral intake once a day for 10 days at ZT1. Cocaine control rats were injected with an equivalent volume of saline (0.9% NaCl), while sucrose control rats received an equivalent volume of water. Rats were anesthetized with 1 mL of Dolethal (100 mg/kg i.p.) prior to being killed 5 h, 10 h, or 15 h after the last treatment. All procedures involving animal care were conducted in compliance with national laws and policies (Council directive 87848, 1987, Service Vétérinaire de la Santé et de la Protection animale, permission 67–165 to J.Z., with the Ministère de l’Education Nationale de l’Enseignement Supérieur et de la Recherche (project permission number APAFIS#2133-20151 00,221 087,072 to P.A.) and with international guidelines (NIH publication 5586-23, 1985). They have also been carried out in accordance with international guidelines on animal experimentation of the Netherlands Institute for Neuroscience (NIN) and with approval by the Animal Care Committee of the Royal Netherlands Academy of Arts and Sciences (KNAW, Amsterdam, The Netherlands).

### 2.2. Brain Dissection and RNA Extraction

Brain structures of interest were dissected (Supplementary Figure S1) and frozen at −80 °C, as previously described) [11]. Total RNA was then extracted from the rat medial prefrontal cortex (PFCx) and the caudate putamen (CPu) and its concentration was evaluated using a Nanodrop spectrophotometer while RNA was stored at −20 °C, as previously described [5].

### 2.3. Reverse Transcription-Quantitative PCR Analysis

Briefly, 0.5 μg of total RNA was reverse transcribed using random primers and Reverse Transcriptase (MLV). The reaction product was used for real time PCR performed with Hot Pol EvaGreen according to the manufacturer’s instructions (Euromedex, Souffelweyersheim, France) with a CFX Connect Real Time PCR detection System (Bio-Rad), as previously described [12]. Primers listed in Table 1 were from Sigma-Aldrich (Saint Louis, MO, USA) and were designed with Primer 3 software. Results were normalized to *36B4* (*RPLP0*) and used as an internal control for gene expression. Cycling conditions were: 95 °C for 14 min, then 45 cycles of 95 °C for 14 s, 60 °C for 18 s, and 72 °C for 18 s. Properties of PCR products were analyzed by melting curve analysis and their expected sizes were confirmed by 2% agarose gel electrophoresis.

### 2.4. Global DNA Methylation Analysis

High molecular weight DNA was extracted from the PFCx and the CPu at ZT16, as previously described [46]. The 5mC levels were evaluated by using a widely used ELISA colorimetric kit (EPIGENTEKTM Global DNA Quantification Ultra Kit, NY, USA) [47]. Absorbance was determined on a microplate reader at 450 nm. The protocol and calculation of DNA methylation were carried on according to the manufacturer’s instructions.

### 2.5. Immunohistochemistry

Animals used for immunohistochemistry studies were perfused intracardially with saline (0.9% NaCl) followed by 4% paraformaldehyde in 0.1 m phosphate buffer, pH 7.4. Brains were rapidly removed and post-fixed in the same buffer overnight at 4 °C, then cryoprotected in 20% sucrose for 48 h at 4 °C prior to being frozen in isopentane at −40 °C and stored at −80 °C. Coronal sections of 30 μm thickness were prepared and stored at −20 °C in free floating cryoprotectant solution. Prior to staining, antigen retrieval was performed by incubating sections in citrate buffer at 90° C for 10 min. Immunohistochemical staining was carried out using Orexin-A goat polyclonal antibody (C-19, Santa Cruz, sc-8070, 1:3000) or Per2 rabbit polyclonal antibody (Millipore, ab2202, 1:2000) or FosB rabbit monoclonal antibody (Cell Signaling Technology, 5G4, cs-2251, 1:2000) also recognizing the ∆FosB truncated isoform. The specificity of primary antibodies was described by the suppliers and by other studies for Orexin-A [5,48], Per2 [49,50] and FosB [51,52]. Floating sections were washed in TBS, at pH 7.6, and incubated in a solution of 3% H_2_O_2_ (30% hydrogen peroxide, Sigma-Aldrich) in TBS for 10 min and rinsed with TBS for 10 min three times prior to their incubation with primary antibodies overnight at 4 °C. Biotinylated secondary antibodies were incubated for 2 h at room temperature (1:400) followed by avidin-biotin complex (ABC Vectastain Kit, Vector Laboratories) for 1 h at room temperature. Sections were then further incubated in diaminobenzidine (0.5 mg/mL, Sigma-Aldrich) and rinsed with TBS to stop the reaction. Tissue sections were mounted onto gelatine-coated slides and dehydrated through a series of alcohol baths. Images of each region of interest were taken using a microscope (Olympus) equipped with a digital camera. Quantifications were performed with ImageJ processing software and the number of positive cells per field was counted and averaged across three sections per animal, as previously described [11,53]. Counting was made twice by an investigator blinded to the identity of the samples and was expressed in cells/mm^2^.

### 2.6. Statistical Analysis

Statistical analyses were conducted via SigmaPlot 12.0 software, a scientific graphing and statistical analysis software package including normality and variance analyses. No statistical methods were used to predetermine the sample size. Normally distributed data were first tested for significance using Student’s t test prior to be analyzed by one- or -two way analysis of variance (ANOVA) followed by Neuwman–Keuls post-hoc test, when requested, as in our previous studies [5,11,12,54]. The initial sample size was *N* = 5 per group and was sometimes reduced to 4 for technical or unexpected concerns. For each ZT, first the ‘Group’ effect between control and experimental animals, treated with either cocaine or sucrose, was evaluated and secondly the ‘Time’ effect in control rats injected with saline or exposed to oral water intake. Significance was set at *p* < 0.05 and data are expressed as means ± S.E.M.

## 3. Results

### 3.1. Chronic Daily Cocaine and Sucrose Administration Does Not Affect Rat Body Weight

**Figure 1:**
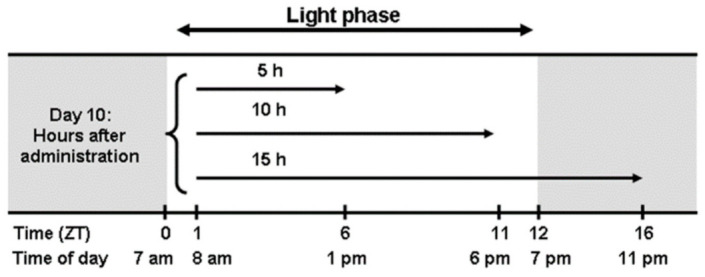
Chronic daily administration of cocaine and sucrose. Daily cocaine and sucrose administration was performed for 10 days at 8 am, 1 h after the beginning of the light phase ranging from Zeitgeber Time 0 (ZT0) = lights on to ZT12 = lights off. After 10 days, rats were sacrificed 5 h, 10 h and 15 h after the last administration at the indicated time denoted as ZT (6, 11 and 16) or time of the day. Zeitgeber time (ZT) is a standardized 24-h notation in which ZT0 indicates the beginning of the light phase and ZT12 is the beginning of the dark phase.

Sucrose reward has been reported to be a stronger reinforcer than cocaine in rodents, as sweetness can surpass cocaine reward, even in drug-sensitized individuals [55,56]. Daily administration for 10 days was performed by i.p. injection for cocaine or saline and by passive oral intake for sucrose and water and animals were sacrificed 5, 10, and 15 h after the last administration corresponding to Zeitgeber Time ZT6, ZT11, and ZT16 (Figure 1) for further daily analyses. Body weight was evaluated in the four groups (Supplementary Figure S2) and was found to vary from about 300 to 400 g during 10 days. At day one, no significant difference was observed between the lowest body weight group (passive water administration) and the highest body weight group (passive saline injections), both differing of 10%. Overall, the weight evolution curves from day 1 to day 10 remained similar in the four groups and at day 10 a difference of 11% was noticed between the same lowest and highest body weight groups, without significant difference. We therefore concluded that there is no relation between reward and body weight in our experimental conditions.

### 3.2. Cocaine and Sucrose Induce Opposite Effects on Dnmt3a and Dnmt3b in the PFCx and the CPu in a Time Dependent Manner

**Figure 2:**
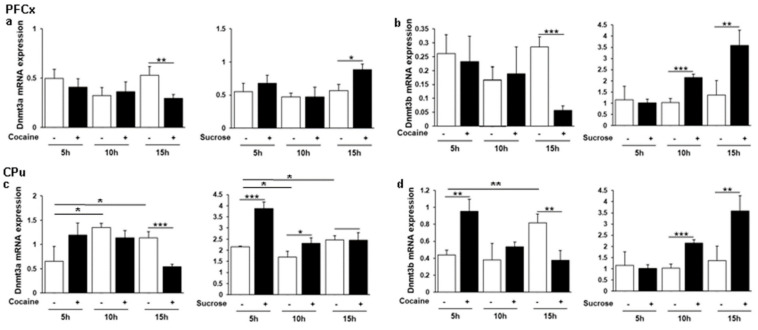
Relative *Dnmt3a* and *Dnmt3b* mRNA expression in the PFCx and the CPu in response to passive cocaine and sucrose intake. Cocaine and sucrose delivery was analyzed in the PFCx (**a**,**b**) and in the CPu (**c**,**d**) by quantitative RT-PCR 5 h, 10 h and 15 h after the last administration, as indicated (corresponding to ZT 6, ZT 11 and ZT 16). *Dnmt3a* expression in the PFCx (**a**) and in the CPu (**c**) was compared with that of *Dnmt3b* in the same brain structures (**b**,**d**), respectively. The amount of each transcript was normalized to that of 36B4. Data represent the mean ± SEM, *n* = 4–5 per group. Statistical analysis performed was one-way ANOVA followed by Newman-Keuls post hoc when required. Significant differences between control and treated groups and within control groups relative to ZT 6 are indicated. * *p* < 0.05, ** *p* < 0.01, *** *p* < 0.001.

Among DNA methyltransferases (Dnmts) catalyzing the transfer of a methyl group predominantly at CpG dinucleotides to generate 5-methylcytosine (5mC), Dnmt3a and 3b are required for de novo DNA methylation [57] and are regulated by cocaine in various brain structures [10,11,14]. Their daily pattern of mRNA expression was measured by RT-qPCR in the PFCx and the CPu of rats following cocaine i.p. injection and sucrose oral administration (Figure 2). In the PFCx (Figure 2a,b), the expression of both *Dnmts* was only affected within the dark phase at ZT16. Indeed, both genes were found to be significantly repressed by cocaine and induced by sucrose intake at ZT16, while no significant effect of time was observed in either group of control rats. Likewise, an opposite pattern was observed in the CPu (Figure 2c,d), with *Dnmt3a* being inhibited by cocaine at ZT16, but induced earlier and transiently by sucrose intake at ZT6 and ZT11. Surprisingly, *Dnmt3b* was transiently induced by cocaine at ZT6, but its level progressively decreased leading to a repression at ZT16, whereas a progressive increase was found in response to sucrose intake (Figure 2d). In cocaine control rats, changes in the baseline levels of both genes were observed notably at ZT11 and ZT16 relative to ZT6 (Figure 2c,d), ZT16 being a time at which an increase was prevented by cocaine (Figure 2c,d). These changes are most likely to be due to the i.p. injections rather than changes in rhythmic expression, since they were not as significant in sucrose control rats after passive water administration. Therefore, an opposite regulation of *Dnmt3a* and *3b* by cocaine and sucrose was observed at ZT16 during the rat active and nocturnal phase after the last administration at ZT1. This time-restricted modification in response to repeated cocaine administration appears consistent with previous kinetic studies having reported their daily variations or biphasic responses to the drug (Anier et al., 2010 [14]; LaPlant et al. [10], 2010; Pol Bodetto et al., 2013 [11]). We concluded that dynamic DNA methylation of *Dnmt3* target sequences should be differentially methylated by a drug of abuse and a natural reinforcer at least at ZT16, even if no stable expression changes are observed over the day.

### 3.3. Daily Regulation of Tet 1, 2 and 3 Gene Expression by Cocaine and Sucrose

**Figure 3:**
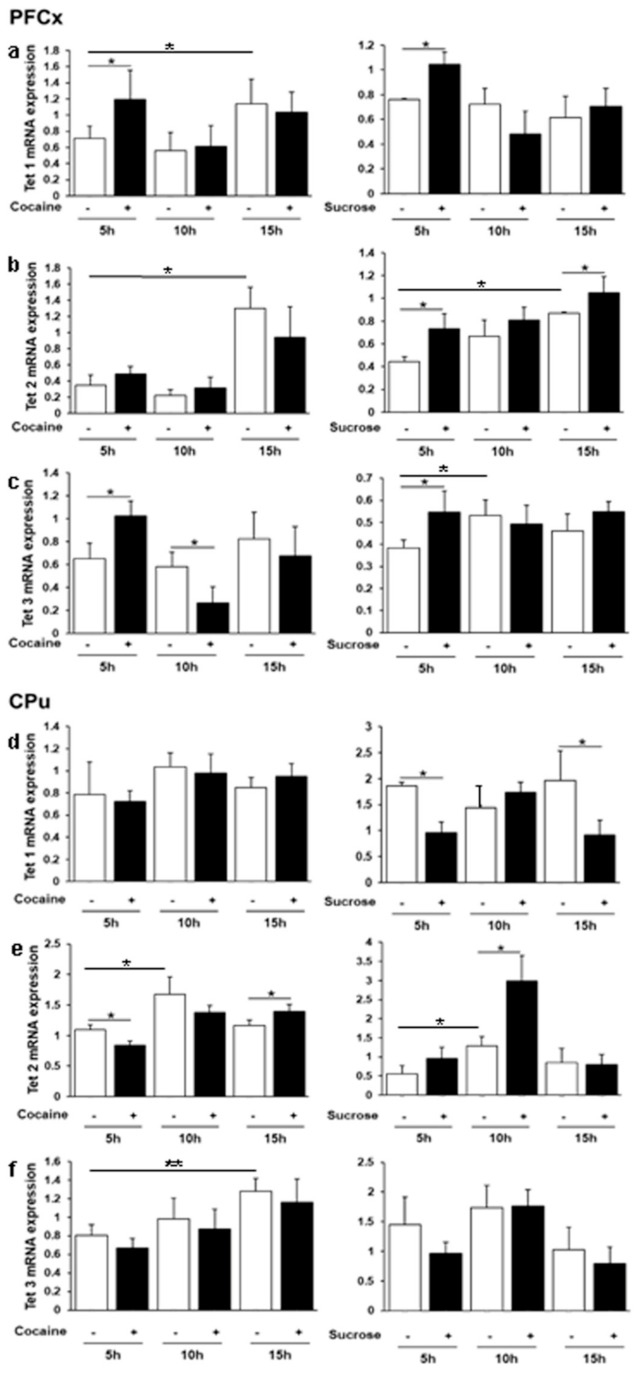
*Tet 1,2* and *3* mRNA expression in the PFCx and in the CPu in response to cocaine and sucrose intake. Relative *Tet 1*, *2* and *3* gene expression was analyzed in the PFCx (**a**–**c**) and in the CPu (**d**–**f**) after the last passive administration at various times corresponding to ZT illustrated in Figure 1. Data represent the mean ± SEM, *n* = 4–5 per group. Statistical analysis performed was one-way ANOVA followed by Newman-Keuls post hoc test when required. Significant differences between control and treated groups and within control groups relative to ZT 6 (5 h after administration) are indicated. * *p* < 0.05, ** *p* < 0.01.

The discovery of *Tet* genes has demonstrated that cytosine methylation is a reversible and dynamic mechanism by which 5mC are oxidized into 5-hydroxymethylcytosine (5hmC), 5-formylcytosine (5fC), and 5-carboxylcytosine (5caC) prior to generate un-methylated cytosines [58,59] involving the base excision repair pathway [60]. In the PFCx and CPu, fewer differences were observed in Tet genes expression in response to both reinforcers especially at ZT16 (Figure 3), as compared with *Dnmts*. In the PFCx, both cocaine and sucrose transiently induced *Tet 1* and *3* expression at ZT6, whereas no significant effect of time was noticed in either sucrose or cocaine control rats (Figure 3a,c). On the other hand, *Tet 2* showed a significant rhythmic expression as ther highest levels were found during the dark phase at ZT16, in both control groups (Figure 3b). While *Tet 2* expression was not at all affected by cocaine, it was progressively induced by sucrose intake, reaching the highest levels at ZT16. In the CPu, *Tet 1* was repressed by sucrose at ZT6 and ZT16 but was not modified in response to cocaine. The two control groups did not show any rhythmic regulation (Figure 3d). In contrast, *Tet 2* basal expression levels were increased at ZT11 in both control groups and this effect was magnified by sucrose, but not by cocaine (Figure 3e). Interestingly, its expression pattern in response to cocaine and sucrose was quite different relative to control levels. A transient repression at ZT6 followed by an induction at ZT16 was observed in response to cocaine, whereas a marked induction was only observed at ZT11 in response to sucrose. *Tet 3* expression was not significantly affected by cocaine and sucrose in the CPu, but was solely increased by NaCl injection at ZT16 (Figure 3f).

### 3.4. Global 5-methylcytosines Analysis

**Figure 4:**
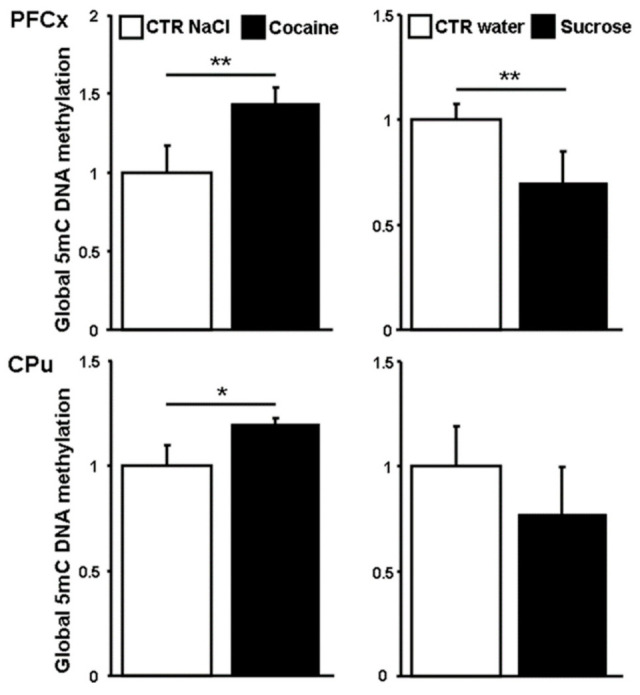
Global 5-methylcytosine analysis in the PFCx and in the CPu. 5mC was measured in response to cocaine and sucrose relative to their controls at ZT16 (15 h after administration). Data represent the mean ± SEM, *n* = 4 per group. Statistical analysis performed was one-way ANOVA followed by Newman-Keuls post hoc when required. * *p* < 0.05, ** *p* < 0.01.

Given the heterogeneous expression of *Dnmt* and *Tet* genes depending on the nature of the genes, the time, and the brain structure considered, we next measured global DNA methylation. The amount of 5mC produced by Dnmt before being oxidized by Tet proteins was evaluated in high molecular weight genomic DNA at ZT16 (Figure 4). In the PFCx and the CPu, a significant 5mC increase of 44% and 20%, respectively, was found in response to cocaine. In contrast, sucrose decreased the amount of 5mC by 30% and 23% in the PFCx and the CPu, respectively.

### 3.5. Effects of Cocaine and Sucrose on Clock and Clock-Controlled Gene Expression

**Figure 5:**
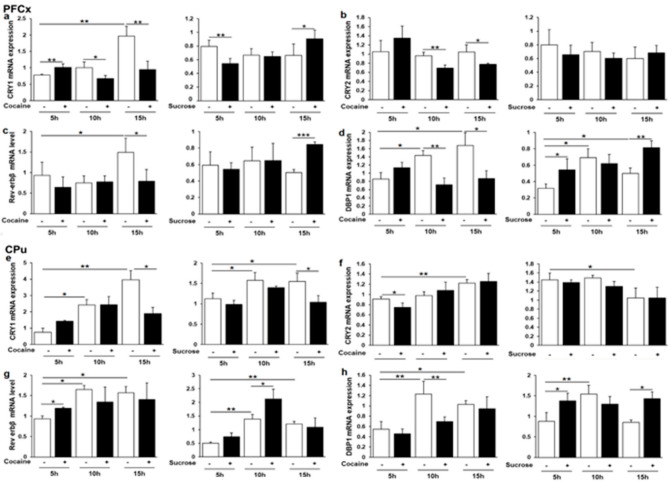
*Clock*, *Bmal1*, *Per1* and *Per2* gene expression in the PFCx and the CPu (**a,b,d–f,h**). Relative mRNA levels (**c,g**) of circadian genes were analyzed as described in Figure 3. Statistical analysis performed was one-way ANOVA followed by Newman-Keuls post hoc test when required. Significant differences between control and treated groups and within control groups relative to ZT 6 (5 h after administration) are also indicated with *n* = 4–5 per group. * *p* < 0.05, ** *p* < 0.01, *** *p* < 0.001.

Clock (Circadian Locomotor Output Cycles Kaput) encodes a transcription factor affecting both the persistence and period of circadian rhythms that orchestrate crucial physiological functions and daily behaviors. It dimerizes with Bmal1 (Brain and Muscle ARNT-like Protein-1) in order to activate other clock-controlled genes such as *Period* (*Per1-3*) and Crypto chromes (*Cry1-2*). Once translated and translocated into the nucleus, the period-cryptochrome protein complex displaces the Clock/Bmal1 heterodimers from chromatin, hence silencing *Per* and *Cry* genes through a transcriptional autoregulatory feedback loop [61]. In the PFCx, *Clock* expression was repressed by cocaine at ZT16 and by sucrose at ZT11. No rhythmic regulation was found in the sucrose control group, whereas its induction in cocaine control rats was likely due to NaCl i.p. injections and was prevented by cocaine (Figure 5a). The regulation of *Bmal1* (Figure 5b), *Per1* (Figure 5c) and *Per2* (Figure 5d) genes by cocaine and sucrose was found to be different at least at one circadian time. *Bmal1* was induced at ZT6 and repressed at ZT11 by cocaine, whereas it was only induced by sucrose at ZT16. A significant rhythmic regulation was observed in sucrose control group with a progressive decrease at ZT11 and ZT16. In contrast, NaCl injections induced its expression at ZT11 and ZT16, this induction was prevented by cocaine at ZT11. *Per1* and *Per2* were both repressed by cocaine at ZT16, whereas they were induced by sucrose at ZT6 and ZT16, respectively. A significant increased expression was observed for both *Per* genes along the day (Figure 5c,d), which was abolished by cocaine, whereas it was further pronounced for *Per2* by sucrose at ZT16.

Similarly, in the CPu, clock gene expression was quite opposite in response to cocaine and sucrose administration relative to control rats (Figure 5e–h). In addition, while a significant rhythmic *Bmal1* and *Per1/2* expression was noticed in cocaine and sucrose control rats, cocaine was found to disrupt or to counteract it, whereas sucrose was found to further emphasize this periodic expression at some ZT (Figure 5f–h). This rhythmic expression is consistent with previous reports having shown a daily regulation by dopamine notably for *Per* genes in the dorsal striatum [62]. We therefore concluded that the two reinforcers markedly differ in regulating major circadian genes expression by their own relative to controls in both dopamine brain projections structures.

### 3.6. Cry1, Cry2, Rev-erbβ and Dbp1 Expression in Response to Cocaine and Sucrose

**Figure 6:**
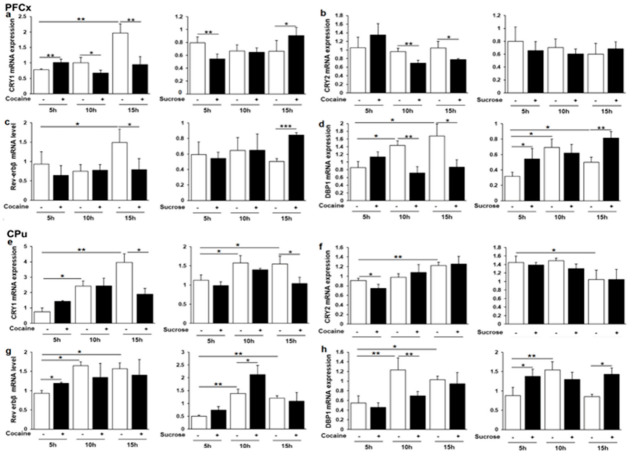
*Cry1*, *Cry2*, *Rev-erbβ* and *Dbp1* mRNA levels in the PFCx and the CPu (**a,b,d–f,h**)**.** Relative mRNA levels (**c,g**) of the 4 genes and statistical analyses were performed, as described in legend to Figure 2. * *p* < 0.05, ** *p* < 0.01, *** *p* < 0.001.

Additional Clock-controlled genes were next analyzed including *Cry1*, *Cry2*, *Rev-erbβ,* and *Dbp1* (albumin D-element binding protein), the later transcription factor being often used as a molecular marker of circadian clock output [63]. In the PFCx, *Cry1*, *Cry2*, *Rev-erbβ*, and *Dbp1* gene expression was found to be different in response to cocaine and sucrose at least at one ZT (Figure 6a–d). In sucrose control rats, only *Dbp1* was found to have a significant rhythmic expression (Figure 6d), the rhythmic expression occasionally observed in cocaine control rats being likely due to the effect of saline i.p. injection [64]. In the CPu, *Cry1* was repressed by both reinforcers at ZT16 (Figure 6e), while a moderate *Cry2* repression was solely observed for cocaine at ZT6 (Figure 6f), both genes exhibiting a rhythmic expression. *Rev-erbβ* was upregulated by both cocaine and sucrose at ZT6 and ZT11, respectively (Figure 6g), whereas an opposite regulation was noticed for *Dbp1* with a significant effect of time at ZT11 (Figure 6h). Overall, the two rewarding agents strongly differ in their effect on clock gene expression in both dopamine projection structures.

### 3.7. Neuropeptide Y and Orexin Regulation by Cocaine and Sucrose

**Figure 7:**
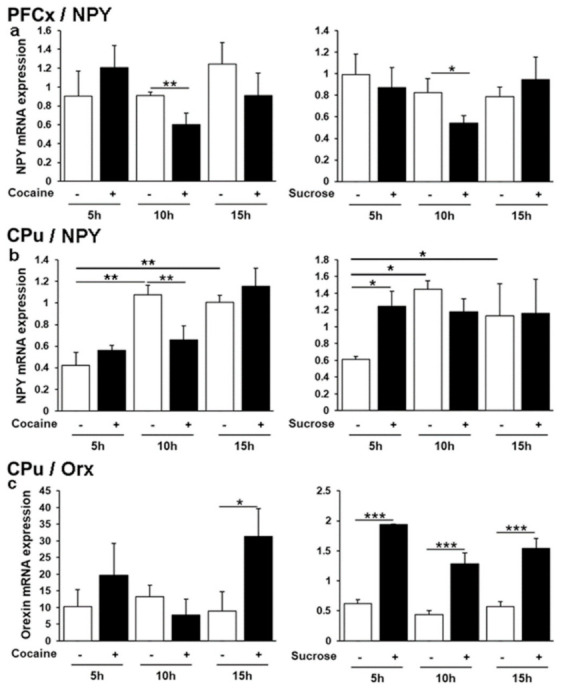
NPY and orexin neuropeptides mRNA levels in the PFCx and the CPu. Relative expression of mRNAs was analyzed(**a–c**), as described in legend to Figure 2. * *p* < 0.01, ** *p* < 0.01, *** *p* < 0.001.

**Figure 8:**
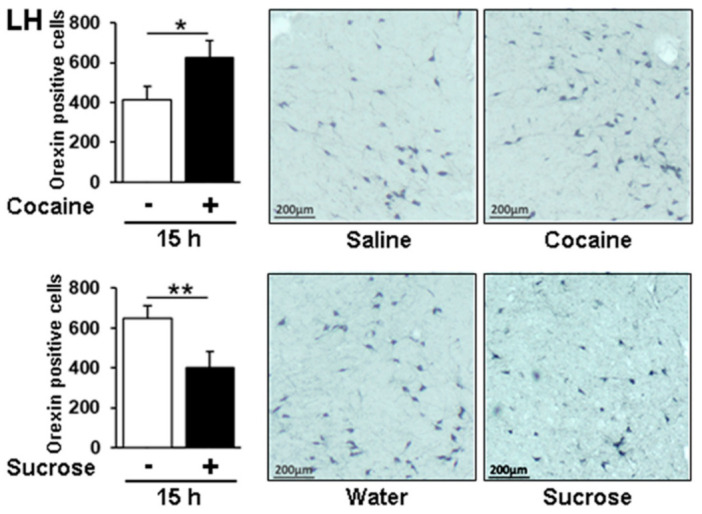
Effect of passive administration of cocaine or sucrose on orexin peptide in the lateral hypothalamus by IHC. Quantification and photomicrographs showing orexin immunoreactivity at ZT 16 in the LH of rats treated daily for 10 days with saline or 20 mg/kg cocaine (one i.p. injection per day) or with sucrose (10 days, one oral administration per day). Statistical analysis performed was one-way ANOVA followed by Newman-Keuls post hoc test. Significant differences are indicated with *n* = 4–5 per group. * *p* < 0.05, ** *p* < 0.01. Scale bar, 200 µm.

Neuropeptide Y (Npy) is widely expressed throughout the central nervous system in which it is co-secreted with classic neurotransmitters, including GABA and glutamate [65]. It plays a crucial role in cortical excitability, stress response, food intake and circadian rhythms [66]. The *Orexin* gene has a more restricted expression pattern, being only transcribed in the lateral hypothalamus (LH), although we have provided evidence for *orexin* mRNA in the CPu, suggesting its transport from the LH [5]. Both Npy and orexin share common functions notably in the regulation of appetite and satiety and their receptors are considered as promising therapeutic targets for addictive behaviors and metabolic diseases [66]. In the PFCx cocaine and sucrose led to a consistent decrease in *Npy* mRNA expression at ZT11, without any significant effect of time in the corresponding control groups (Figure 7a). In contrast, the two control groups showed a significant upregulation at ZT11 and ZT16 in the CPu in which cocaine was preventing the time effect at ZT11, whereas *Npy* was solely induced by sucrose at ZT6 highlighting a reward agent specific response (Figure 7b). No effect of time was found for *orexin* mRNA levels in the CPu, suggesting that mRNA transport from the LH is not regulated in a time-of-day dependent manner. The transport was increased by cocaine at ZT16, whereas mRNA levels were much higher and striking compared with controls after sucrose administration at all time points (Figure 7c). Orx expression was further analyzed at ZT16 by IHC in the LH where it is synthesized (Figure 8). The number of Orx-positive cells was found to be higher in response to cocaine and lower in response to sucrose. The data therefore provide additional evidence concerning how the effects of a drug of abuse and a natural reinforcer differ in modulating genes at the protein level following repeated administrations.

### 3.8. Opposite Pattern of Per2 Positive Cells in Response to Sucrose and Cocaine in the LH

**Figure 9:**
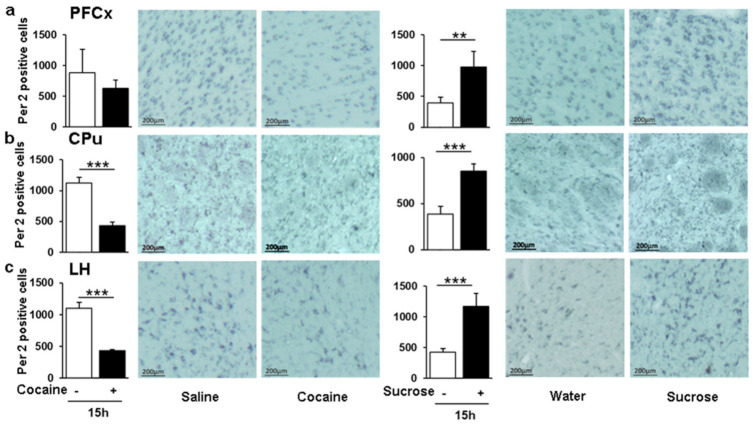
Per2 analysis by IHC in response to passive cocaine and sucrose administration. Quantification and photomicrographs showing Per2 immunoreactivity in the PFCx, CPu and LH of rats treated daily for 10 days with saline or 20 mg/kg cocaine (one i.p. injection per day) and with sucrose (one administration per day) (**a**–**c**). Statistical analysis performed was one-way ANOVA followed by Newman-Keuls post hoc test. Significant differences are indicated with *n* = 4–5 per group. ** *p* < 0.01, *** *p* < 0.001.

We next examined Per2 expression by IHC at ZT16 in the PFCx, the CPu, and the LH (Figure 9a–c). Strikingly, in all brain structures, the number of positive cells was lower in cocaine treated rats and higher in rats receiving sucrose relative to controls. It is noteworthy that the data parallel those obtained for mRNA in response to each reinforcer (Figure 4d,h). At higher magnification, Per2 cellular staining was diffuse with nuclear and cytosolic localization, consistent with previous reports [67]. The number of positive cells in NaCl i.p. injected rats was higher than that in rats receiving water orally (Figure 9), indicating that repeated injections by themselves also modulate Per2 expression, although these changes did not correlate with those found at the mRNA levels at ZT16 (Figure 4d,h). Nevertheless, that Per2 positive cells are down-regulated by cocaine and up-regulated by sucrose underlines some specific mechanisms different for each rewarding agent.

## 4. Discussion

The present study demonstrates that the response to cocaine and sucrose of many genes in dopamine projection areas and the hypothalamus is regulated in a time-dependent manner. While interactions between circadian rhythms and drugs of addiction within the mesocorticolimbic reward system have been reported previously [28,68,69,70], our findings show that a drug of abuse and a strong natural reinforcer in rodents trigger different changes in gene and protein expression in these brain areas. The genes and proteins investigated as part of mechanisms controlling DNA methylation, appetite, satiety, and circadian rhythms, underline that, depending on the reinforcer type, alterations in critical biological functions and their behavioral consequences are different. The current data further support initial fMRI and electrophysiological studies [1,2,3] and provide new insights into molecular and cellular mechanisms showing that the neural rewarding circuits activated by both reinforcers do not overlap.

Drugs of abuse and natural reinforcers can lead to addiction characterized as complex relapsing disorders occurring overtime in which compulsive seeking behavior can persist despite aversive consequences. Dopamine is known to play an essential role in the motivational aspect of reward and the dopamine theory of addiction highlights its major and common role in the mesolimbic system [71,72]. That is why we have looked at dopamine projection areas (PFCx and CPu), although drugs of abuse also sensitize noradrenergic and serotonergic neurons via non-dopaminergic mechanisms [6,69,73,74] and rewarding drugs, such as opiates or psychostimulants, induce different behavioral and neurobiological responses [75,76]. Developing pharmacological interventions specifically targeting dopamine receptors or its transporter system has also been reported to be a daunting task for the treatment of drugs of abuse [77]. Thus, the unitary account of addiction involving dopamine [76] has been challenged by several studies.

Here, we have compared chronic passive cocaine intake with that of sucrose since the latter is the main source of energy in the brain and a major component in highly palatable food. While sweetness has been reported to surpass cocaine reward in rodents [55], sugar addiction has been the subject of controversial issues [78,79]. Contrary to cocaine, little evidence has been provided for human sugar addiction or for a sugar addiction model of overweight to support clinical DSM criteria, suggesting that sugary food does not directly promote excessive weight or obesity, but rather contributes minimally to ‘food dependence’ and increased risk of weight gain by overeating [80,81]. By analyzing body weight development, no significant differences were found between cocaine and sucrose treated rats and their respective control groups (Supplementary Figure S2).

Overlap between drugs of abuse and natural reinforcers has been well illustrated by considering orexin, NPY, and ghrelin. Indeed, these peptides regulate appetite and satiety and their receptor antagonists have been characterized as promising therapeutic targets for addictive behaviors, drug abuse disorders, or metabolic diseases [38,39,40,65,82]. Orexinergic neurons widely project throughout the brain, including key structures of the limbic system, which are “multi-tasking” neurons regulating functions like arousal, sleep/wake states, feeding behavior, energy homeostasis, anxiety, and addictive behaviors. *Orexin* mRNA was induced by both cocaine and sucrose in the CPu although with a different timing (Figure 6c). In the LH, i.e., its site of transcription, the number of orexin positive cells was increased by cocaine but decreased by sucrose 15 h after the last administration (Figure 7, ZT16). This increase elicited by cocaine is in line with the upregulation of the number of LH orexin neurons after exposure to various drugs of abuse [83] and with the critical role of orexins in controlling arousal [84,85] and in the motivation for cocaine notably illustrated in Orx and Orx R1 knockdowns [37,86]. Moreover, it is noteworthy that the *Orx R1* gene has been shown to be regulated by cocaine through DNA methylation [5].

Variations in *Dnmt* and *Tet* gene expression in response to reinforcers have been documented in various experimental models. *Dnmts* display biphasic time-course regulation following cocaine chronic passive treatment or in cocaine self-administering rats [5,10,11,14]. Tissue specific biological functions and specific target sequences have been reported for both gene families [87,88,89]. Our data concerning cocaine treatment are consistent with these studies, showing a heterogeneous pattern of expression depending not only on the brain structures, but also on the time of the day considered (Figure 2 and Figure 3). We also found a significant increase in global DNA methylation following cocaine administration and a decrease in response to sucrose in the PFCx and the CPu (Figure 4), while other global DNA methylation analyses have reported no or little changes in response to cocaine [33,34,36,90,91,92].

To some extent, chronic passive and voluntary drug intake cause similar behavioral and molecular effects. We therefore looked for differentially methylated regions (DMRs) associated with the 15 genes analyzed here from a genome-wide study performed in the PFCx of cocaine self-administering rats [36]. DMRs were found for 13 genes studied here, suggesting that they are regulated by DNA methylation. In agreement with previous global analyses, most DMRs were found within gene bodies for which no obvious correlation was established between 5mC hypermethylation and gene repression. Nevertheless, a single hypermethylated DMR was identified upstream at −2419 bp relative to the transcription start site of the *Clock* gene with a methylation ratio (cocaine)/(saline) of 1.44, that was repressed by 40% at ZT16. In addition, an upstream DMR at −2719 bp relative to the transcription start site of the *Dbp1* gene was repressed by 52% at ZT11, while it was found to be hypermethylated with a 5mC ratio (cocaine)/(saline) of 1.18. The *Cry2* gene that is repressed at ZT11 and ZT16 by 36 and 26%, respectively, was found to be hypermethylated in a downstream sequence with a 5mC ratio (cocaine)/(saline) of 1.75. Considering that cocaine and sucrose differentially regulate the expression of *Dnmt* and *Tet* genes (Figure 2 and Figure 3) as well as global DNA methylation (Figure 4) in the PFCx and the CPu, one may speculate that both agents consequently differ in modulating the rhythmicity of day/night cycles by altering gene expression resulting from chromatin modification in the brain.

While the role of orexins in the central and peripheral regulation of glucose homeostasis and metabolism has been reported, the effect of sugar on orexin expression or production has been less documented. Nevertheless, hypothalamic orexin neurons regulate arousal according to energy balance and a negative correlation was found between orexin expression and blood glucose and food intake [93]. Glucose was also reported to inhibit orexin neurons [94,95], and conversely, glucodeprivation was activating them [96]. In this respect, the decrease in orexin positive cells found in the LH following sucrose passive intake (Figure 7) further illustrates a marked difference relative to cocaine administration. In the CPu, sucrose was found to induce orexin mRNA levels (Figure 6c), which may sound surprising relative to the decreased orexin peptide expression in the LH (Figure 7). However, orexins display local brain structure-specific functions, so such differences may be attributed to different regulatory mechanisms between peptide exclusively transcribed in the LH and its mRNA transport to the CPu recently documented [5].

Unlike orexins, Npy neuropeptide is widely expressed throughout the central nervous system and secreted along with GABA and glutamate [65]. Because of its widespread distribution, including the peripheral nervous system, it is implicated in multiple physiological processes like cortical excitability, stress response, food intake, energy metabolism, memory, sleep regulation, and circadian rhythms [53]. While its regulation in the CNS has been studied extensively in the hippocampus and the hypothalamus, its expression in response to cocaine relative to sucrose has not yet been well investigated in the PFCx and CPu. In the PFCx, its baseline expression was not affected at any time, and it was solely repressed at ZT11 by both cocaine and sucrose (Figure 7a). This similar regulation suggests a common role of both reinforcers in NPY-mediated cortical cognitive functions such as short term/working or maintenance of memory, attention, and arousal. In the CPu, *Npy* showed a time-of-day dependence in both control groups (Figure 7b), indicating that, in this brain structure, unlike other genes, its expression is not solely affected by daily NaCl injections which may be associated with stress or pain [64] that can also affect DNA methylation in male rats notably following nerve injury [97].

As expected, analyzing all genes, independently of those only regulated by circadian rhythms, revealed a high number modulated by both reinforcers (Supplementary Figure S3). Indeed, most genes were induced by sucrose in the PFCx (10 out of 14) and in the CPu (10 out of 15), whereas most of them were repressed by cocaine in the PFCx (9 out of 14) and the CPu (8 out of 15). Hence, sucrose and cocaine strongly differ in the regulation of 11 out of 14 genes in the PFCx and 10 out of 15 in the CPu at least at one time point. The overall data further confirm earlier functional magnetic resonance imaging [1], as well as electrophysiological [3,16] and molecular studies [5,11] by showing that neural circuits activated by cocaine and natural reinforcers do not overlap.

In summary, our data highlight a discrepancy in the molecular and cellular mechanisms triggered by sucrose and cocaine in dopamine projection areas and in the hypothalamus. We previously reported that various genes were differentially affected by the voluntary administration of food vs. cocaine [5,11]. By analyzing key factors involved in DNA methylation, circadian rhythms, and appetite and satiety in passive intake, we provide new insights into marked differences in mechanisms occurring in the brain upon the administration of a drug of abuse or a natural reinforcer independently of the mode of administration. Whether the described cocaine-induced changes are long lasting remains to be investigated, but cocaine and non-natural reward were shown to produce persistent LTP in the VTA [98] and persistent variations in neuronal DNA methylation [99]. In addition, as recently reviewed, some studies have documented long-lasting changes in the expression of epigenetic enzymes and molecules that persist for weeks after the last drug exposure [100].

The regulation of DNA methylation factors by both agents raises questions regarding their common and tissue-specific target-methylated DNA sequences [88,101,102] and their role in the establishment and development of drug addiction. Cocaine alters the epigenome with potential consequences on future generations and de novo DNA methylation changes can be inherited in acquired or innate behaviors or diseases induced by environmental factors [14,103,104,105,106]. Understanding the mechanisms dissociating drugs of abuse from natural reinforcers is a prerequisite for the design of selective therapeutic tools to treat compulsive behaviors or addictions in the fascinating field of neuroepigenetics.

## Figures and Tables

**Table 1:** Primer sequences.

Gene	Primer sequence (5′–3′)	GeneBank Acc. #
*Dnmt3a*	FP:GCTGAAGGAGAGGGAACTGA RP:TGCCTGGAAGGTGAGTCTTG	NM_001003958
*Dnmt3b*	FP:TGCGGTAAGAAGAACCCTGT RP:CTGATAGCCGTCCTCATCGT	NM_001003959
*Tet1*	FP: CTGTGGGGAATGCACCTACT RP: TGGCTTCTTTTTGAGCACCT	XM_008774952.1
*Tet2*	FP: AGAAGCGTAAGAAGCGCAGT RP:TCTTTTTCATTTGACCGTCTCTTCC	XM_006224264.2
*Tet3*	FP: TGTGTGCAAGAGGACTTTCG RP: TACTGACGGGTGGTTTCTCC	XM_006224966
*CLOCK*	FP: GTCATCCTTCAGTAGTCAGTCCA RP: ACATGCCTTGTGGGATTGGT	NM_021856.2
*Bmal 1*	FP: ACTGTCTAGGTGGAGGATTTTGG RP: CTGGTCACCTCAAAGCGACT	NM_024362.2
*Per1*	FP: TACCAGCCATTCCGCCTAAC RP: CGGGGAGCTTCATAACCAGA	NM_001034125.1
*Per2*	FP: CACCCTGAAAAGAAAGTGCGA RP: CAACGCCAAGGAGCTCAAGT	NM_031678.1
*Cry1*	FP: AGGACGCACAGAGTGTTGG RP: TCCTCCCGCATGCTTTCGTATC	NM_198750.2
*Cry2*	FP: GGGGACTACATCCGGCGATA RP: ATGATGCACTTAGCGGCCTT	NM_133405.2
*Rev-erb* *β*	FP: GGGAGGATGCATCTGGTTTG RP: CACCTCTTTTACTGCTGGGG	NM_147210.2
*NPY*	FP: TGGCCAGATACTACTCCGCT RP: GCTGGATCTCTTGCCATATCTCT	NM_012614.2
*DBP1*	FP: AAGGCAAGGAAAGTCCAGGT RP: TGGCTGCTTCATTGTTCTTG	NM_012543.3
*Orx*	TCCTTGGGTATTTGGACCAC CCCAGGGAACCTTTGTAGAAG	NM_013179
*36B4*	FP:GTGCCTCACTCCATCATCAA RP:TCCGACTCTTCCTTTGCTTC	NM_022402

Primer sequences used for real time qPCR are indicated with gene names and GenBank/NCBI accession numbers (#). FP and RP indicate forward and reverse primers, respectively.

## Data Availability

Not applicable.

## Supplementary Materials

The following are available online at www.mdpi.com/article/10.3390/genes12081195/s1, Figure S1: Dissected brain regions for mRNA analysis, Figure S2: Rat body weight during daily cocaine and sucrose administration, Figure S3: Overall gene regulation by sucrose and cocaine relative to controls.
