# Supplementary material for: Rhythmic Regulation of DNA Methylation Factors and Core-Clock Genes in Brain Structures Activated by Cocaine or Sucrose: Potential Role of Chromatin Remodeling
Source: Genes (Basel). 2021 Jul 31;12(8):1195. doi: 10.3390/genes12081195 (PMC8392220; doi:10.3390/genes12081195)
Supplement: Supplementary file 1 [file genes-12-01195-s001.zip › genes-1307841-supplementary.pdf]

Supplemental Materials

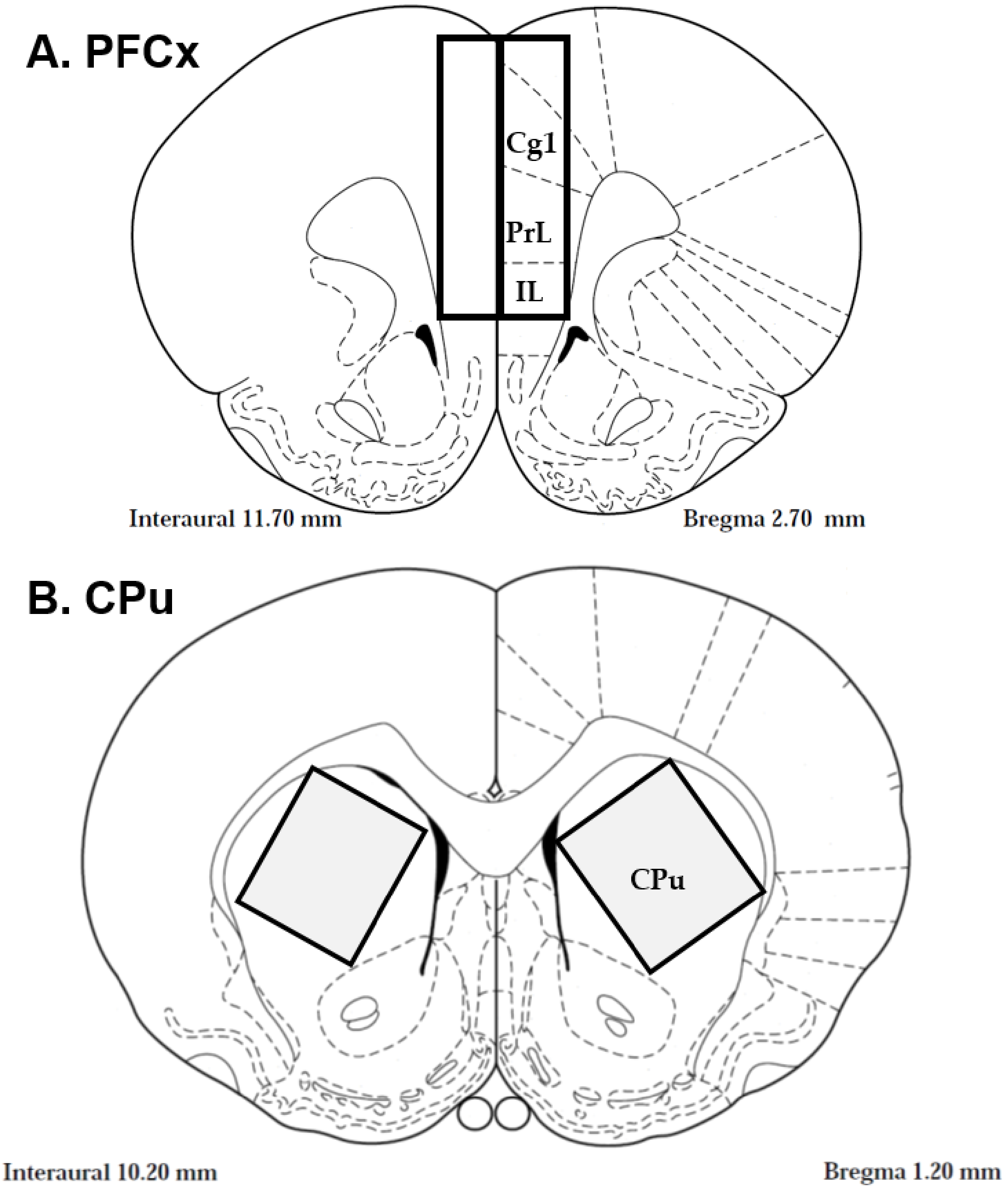

C1. Hypothalamus, rostral side of the section

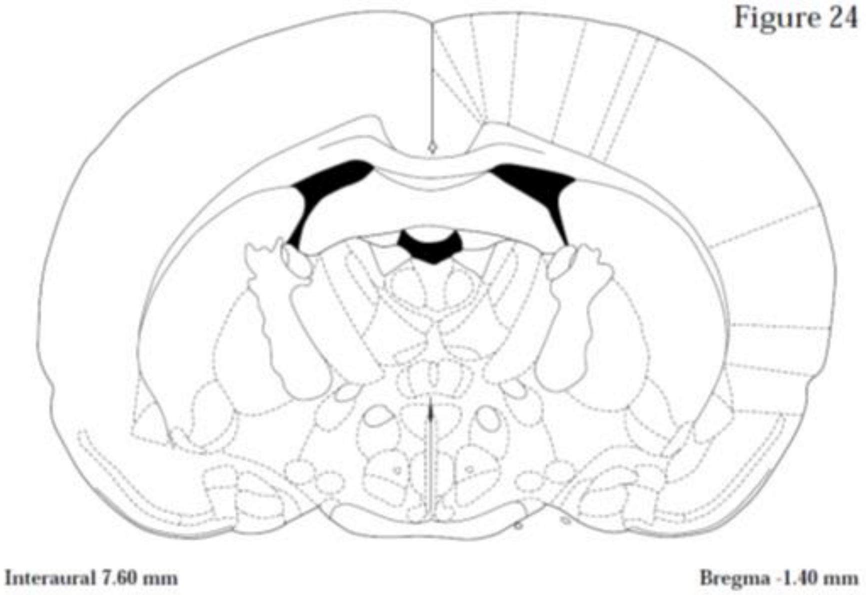

C2. Hypothalamus, caudal side of the section

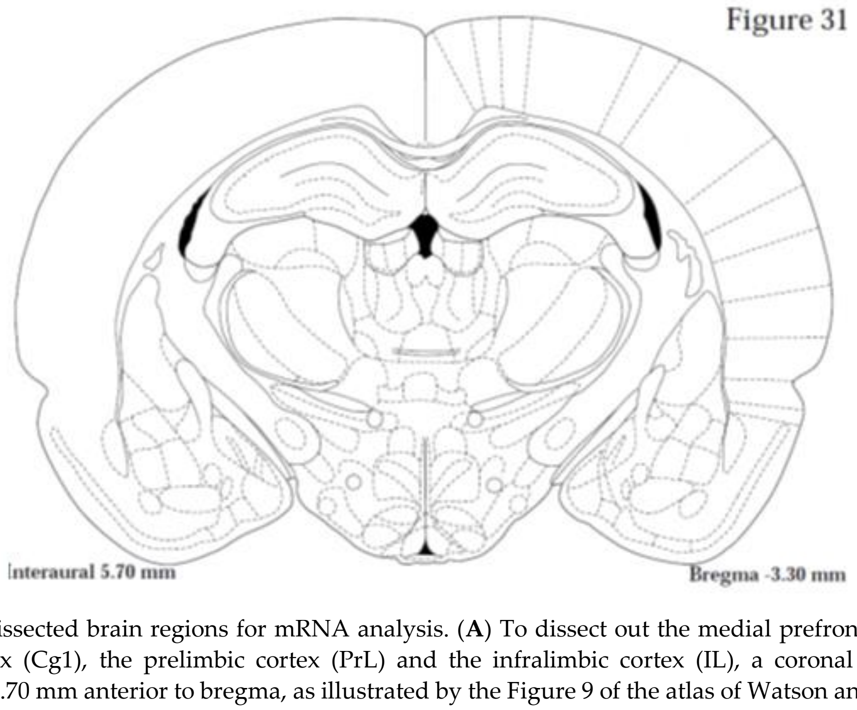

Supplementary Figure S1. Dissected brain regions for mRNA analysis. (**A**) To dissect out the medial prefrontal cortex, including the cingulate cortex (Cg1), the prelimbic cortex (PrL) and the infralimbic cortex (IL), a coronal slice was performed approximately at 2.70 mm anterior to bregma, as illustrated by the Figure 9 of the atlas of Watson and Paxinos (Paxinos, 2007). The medial part of the brain located medially to the corpus callosum was collected, as indicated by 2 rectangles. (**B**) The caudate-putamen (CPu/shadowed areas) was dissected out from a 1.5 mm width coronal section beginning from the previous slice and ending approximately 1.2 mm anterior to the bregma, as illustrated by the Figure 13 of the atlas of Watson and Paxinos. (**C**) Hypothalamus was dissected from the rostral side (**C1**) to the caudal side (**C2**), as indicated by areas marked with a blue borderline. Dissected brain regions for mRNA analysis. (**B**) To dissect out the medial prefrontal cortex, including the cingulate cortex (Cg1), the prelimbic cortex (PrL) and the infralimbic cortex (IL), a coronal slice was performed at 2.70 mm anterior to bregma, as illustrated by the Figure 9 of the atlas of Watson and Paxinos

(Paxinos, 2007). The medial part of the brain located medially to the corpus callosum was collected, as indicated by 2 rectangles. (**B**) The caudate-putamen (CPu/shadowed areas) was dissected out from a 1.5 mm width coronal section beginning from the previous slice and ending approximately 1.2 mm anterior to the bregma, as illustrated by the Figure 13 of the atlas of Watson and Paxinos. (**C**) Hypothalamus was dissected from the rostral side (**C1**) to the caudal side (**C2**), as indicated by areas marked with a blue borderline. Paxinos GW, C. (2007) The rat brain in stereotaxic coordinates. 6th edn Academic Press, Elsevier.

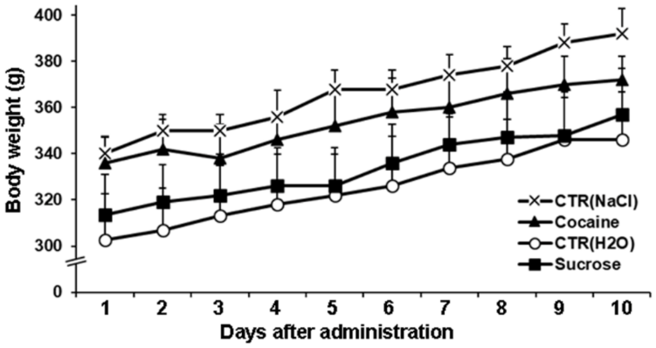

SupplementaryFigure S2. Rat body weight during daily cocaine and sucrose administration. Daily cocaine and sucrose administration was performed for 10 days at 8 am, 1 h after the beginning of the light phase ranging from Zeitgeber Time 0 (ZT0) = lights on to ZT12 = lights off. Everyday, rat body weight was measured after reinforcer administration relative to control rats (CTR). No significant difference was observed between the 4 groups. Data represent the mean ± S.E.M., n = 4–5 per group. Statistical analysis performed was one-way ANOVA.

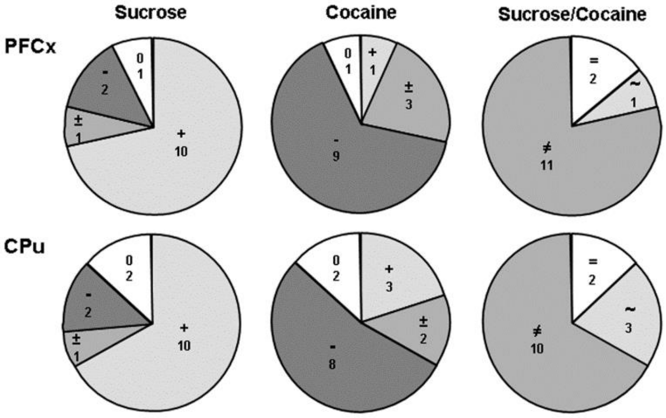

SupplementaryFigure S3. Overall gene regulation by sucrose and cocaine relative to controls. In the four first diagrams on the left, genes are distributed in 4 sections depending on their regulation by sucrose and cocaine relative to their corresponding controls (saline and water, respectively) in the PFCx and in the CPu, as indicated. Gene expression was either induced (+), or induced and repressed (±), or repressed (-) at least at one ZT or remained unaffected (0) by the reinforcers. The comparison between sucrose and cocaine is depicted in the two diagrams on the right in which sections indicate either an identical (=), a similar (~) or an opposite (≠) regulation. In all diagrams, the number indicated below the type of regulation refers to the corresponding number of genes in each section, as evaluated in other figures (2, 3 and 5 to 7).
